# The incidence and cost of devices explanted during single-level anterior diskectomy/fusions

**DOI:** 10.4103/2152-7806.77033

**Published:** 2011-02-23

**Authors:** Nancy E. Epstein, Garry S. Schwall, Donald C. Hood

**Affiliations:** 1 Department of Neurological Surgery, The Albert Einstein College of Medicine, Bronx, NY, USA; 2 Neurosurgical Spine and Education, Winthrop University Hospital, Mineola, NY, 11501, USA; 3 Long Island Neurosurgical Associates, New Hyde Park, NY 11042, USA; 4 Chief Operating Officer, Winthrop University Hospital, Mineola, NY 11501, USA; 5 Department of Psychology, Columbia University, New York, NY 10027, USA

**Keywords:** Anterior diskectomy/fusion, costs, explanted devices, instrumentation, single-level

## Abstract

**Background:**

Little is known about the costs of devices explanted during anterior cervical diskectomy and fusion surgery. This retrospective study analyzes the costs to a single hospital of plates, screws and spacers used in all single-level anterior diskectomy and fusion (single-ADF) operations performed during a 1-year period.

**Materials and Methods:**

Our objective was to determine the costs of instrumentation explanted (i.e. implanted during surgery but removed prior to closure) during 87 single-ADF procedures performed at a single institution within a single year, 2009. All 87 single-ADF procedures were analyzed to determine the frequency and costs (without overhead) to the hospital for both permanently implanted and explanted anterior cervical screws, plates, and spacers (allograft, artificial plastics, and cages). All patients undergoing single-ADF were included in this study irrespective of the diagnosis related group (DRG) category.

**Results:**

The costs, without overhead to the hospital, for the permanently implanted instrumentation were: screws ($103,572: 84 patients); plates ($120,694: 85 patients); allograft spacers ($92,776: 64 patients); cages ($38,821: 9 patients); and autografts (no charge; 14 patients), for a total of $355,863. The additional costs to the hospital for explanted instrumentation were: 37 screws ($11,014: 17 patients); 7 plates ($12,743: 5 patients); and 8 allograft spacers ($9093: 7 patients); there were no explanted cages. The total cost of the explanted devices was $32,850.

**Conclusions:**

During 87 single-ADF procedures, a total of 37 screws, 7 plates, and 8 spacers were explanted in 24 (27.6%) patients, resulting in an additional $32,850, 9.2%, to the cost of the implanted devices.

## INTRODUCTION

The multiple plate/screw systems and bone spacers which have been developed for anterior cervical surgery can be combined resulting in a large number of choices. Although select studies have evaluated the costs of these combined implanted devices, little is known about the frequency and costs associated with explantation (i.e. devices initially implanted, but removed prior to wound closure). The purpose here was to look more closely at the instrumentation used during 87 single-level anterior cervical diskectomy/fusion (single-ADF) operations performed in 2009 at a single institution. In particular, we evaluated the frequency and costs of implanted and explanted devices used to perform these 87 single-ADF procedures.

## MATERIALS AND METHODS

During the calendar year 2009, 87 single-ADF procedures were performed at a single institution. Patients from all DRG categories undergoing single-ADF were included in this study.

We evaluated the cost to the hospital, without overhead, for all instrumentation (i.e. screws, plates, and spacers (allograft, cages, and autograft)) utilized in each case. In particular, we not only evaluated the costs of permanently implanted devices, but also calculated the incidence of, and costs associated with, explanted instrumentation. In all cases, the costs incurred by the hospital to the vendors were recorded; associated overhead costs were not included in this study.

## RESULTS

The total cost incurred by the hospital for permanently implanted plates, screws, and spacers utilized to perform the 87 single-ADF was $355,863. Table 1 shows the breakdown by the category. The 85 permanently implanted plates cost $120,694, with the cost of individual plates ranging from $441 to $2025. The screws required to implant each plate ranged in cost from $228 to $457 per screw, and the total cost of the four implanted screws in 85 cases was $103,572. Allograft spacers utilized in 64 cases ranged in cost from $843 to $2552, for a total cost of $92,776. The cost of cages (including top/bottom attachments) ranged from $1720 to $7928; the nine cages cost $38,821. There was no instrumentation cost associated with the iliac autograft utilized in 14 cases.

**Table 1:** Numbers and costs (without overhead) of permanently implanted instrumentation

	Plates	Screws	Spacers (Allograft)	Spacers (Cages)	Spacers (Autograft)
Number of implants	85	336^a^	64	9	14
Number of patients	85	84^a^	64	9	14

a Four screws × 84 patients; in one patient old screws were reused in the operation done in 2009.

The total cost of the explanted instrumentation, including screws, plates, and spacers, was $32,850 or 9.2% of the cost of the implanted instrumentation. The total cost for seven plates, explanted in five cases, was $12,743 [Table 2]. A total of 37 screws were explanted in 17 cases; the number of explanted screws ranged from 1 to 10. The total cost of the explanted screws was $11,014. The total cost for the eight explanted spacers, utilized in seven cases, was $9093. There were no explanted cages reported.

**Individual Surgeons and Explantation** The 87 single-ADF operations were performed by 11 surgeons, with five of these surgeons performing four or fewer operations. The other six surgeons performed between 7 and 25 single-ADF, and accounted for 76 of the 87 operations. All six of these surgeons explanted devices, and explantation occurred in 12.5–46.2% of their cases.

**Table 2:** Number and costs (without overhead) of explanted instrumentation

	Plates	Screws	Spacers (allograft)
Number of explants	7	37	8
Number of patients	5	17	7
Total cost ($)	12,743	11,014	9,093

## DISCUSSION

Devices were explanted in 24, or 27.6%, of the 87 single-ADF performed at one institution during 2009. Explantation was common among the surgeons performing the larger number of cases. For the six surgeons performing 76 of the cases (range 7–25 cases per surgeon), devices were explanted in between 12.5% and 46.2% of their cases. The cost, without overhead, to the hospital for all explanted instrumentation, in all 87 cases, came to $32,850 or 9.2% of the total cost of $355,863 of the permanently implanted instrumentation.

What is an acceptable explantation rate? We were not able to find literature that addresses this important question. In the senior author’s experience, 5–10% of the cases might require explantation of instrumentation. If we assume a target of 10% frequency for explantation, as opposed to the 27.6% observed here, then it should be possible to at least reduce the costs associated with explantation by 50%.

Because of the escalating costs for health care delivery, it is essential to identify opportunities for cost savings. In fact, the costs of explanted devices in single-ADF may underestimate the potential for savings associated with explantation in general. For example, much higher explantation costs might be anticipated for the more extensive cervical, thoracic, and lumbar spinal procedures.

Given the range of costs associated with implanted devices, insurance carriers may soon seek to cap reimbursements to hospitals. Therefore, the benefits of costly implanted spinal devices must be weighed. Unfortunately, this study did not include outcome data. However, a few series of single-ADF have focused on cost-effectiveness. One study examined the relative costs for four treatment groups: the cage alone group was the least expensive, the disc arthroplasty and cage/plate/bone substitute groups showed comparable intermediate costs, while the autograft group was the most expensive; pain at the hip graft site for the latter group required a longer (average 5 day) length of hospital stay (LOS).[1] A second single-ADF study showed similar costs for titanium surgical mesh/local autograft bone/plate (27 patients) compared with iliac bone graft/plate (27 patients).[3] The operative time saved by avoiding iliac bone graft harvesting proved equivalent to the increased cost of the cage. In a previous study, we found that the average operative time for six surgeons performing single-ADF utilizing allograft/cages and plates was 3.6 h with an average LOS of 2.2 days.[4] In comparison, in the first author’s series of 60 patients undergoing single-ADF utilizing only iliac autograft with plates, the average operative time was 3.4 h, nearly comparable, but the average LOS (3.2 days) was one day longer.[5] However, the cost associated with the additional one day is typically less than the costs of the allografts ($843 to $2552) and cages ($1720 to $7928). In two other single-multilevel ADF series, bone morphogenetic protein (BMP), utilized to supplement anterior diskectomy and fusion constructs, resulted in an increased incidence of acute postoperative swelling/dysphagia (50% with BMP, 14% without), which increased hospital costs secondary to prolonged LOS.[2,6] It is clear from these studies that the least expensive constructs are not necessarily the most cost effective, nor are the most expensive necessarily the least cost effective.

In summary, we found that explantation occurred in 27.6% of the 87 cases in this series. This added 9.2% to the cost of implanted devices in single-ADF procedures. Identification of these additional costs has prompted measures to examine and reduce costs associated with explantation in general at our institution.
